# Supplementary material for: Yiai Fuzheng decoction inhibits triple-negative breast cancer by remodeling the immune microenvironment
Source: Front Immunol. 2025 Sep 30;16:1615631. doi: 10.3389/fimmu.2025.1615631 (PMC12518410; doi:10.3389/fimmu.2025.1615631)
Supplement: Supplementary file 2 [file DataSheet1.zip › Supplementary File 2/Treatment/C_vs_D/07_Pathway_Analysis/Pathway_Analysis/Sig_Pathways_Name/Amino sugar and nucleotide sugar metabolism.pdf]

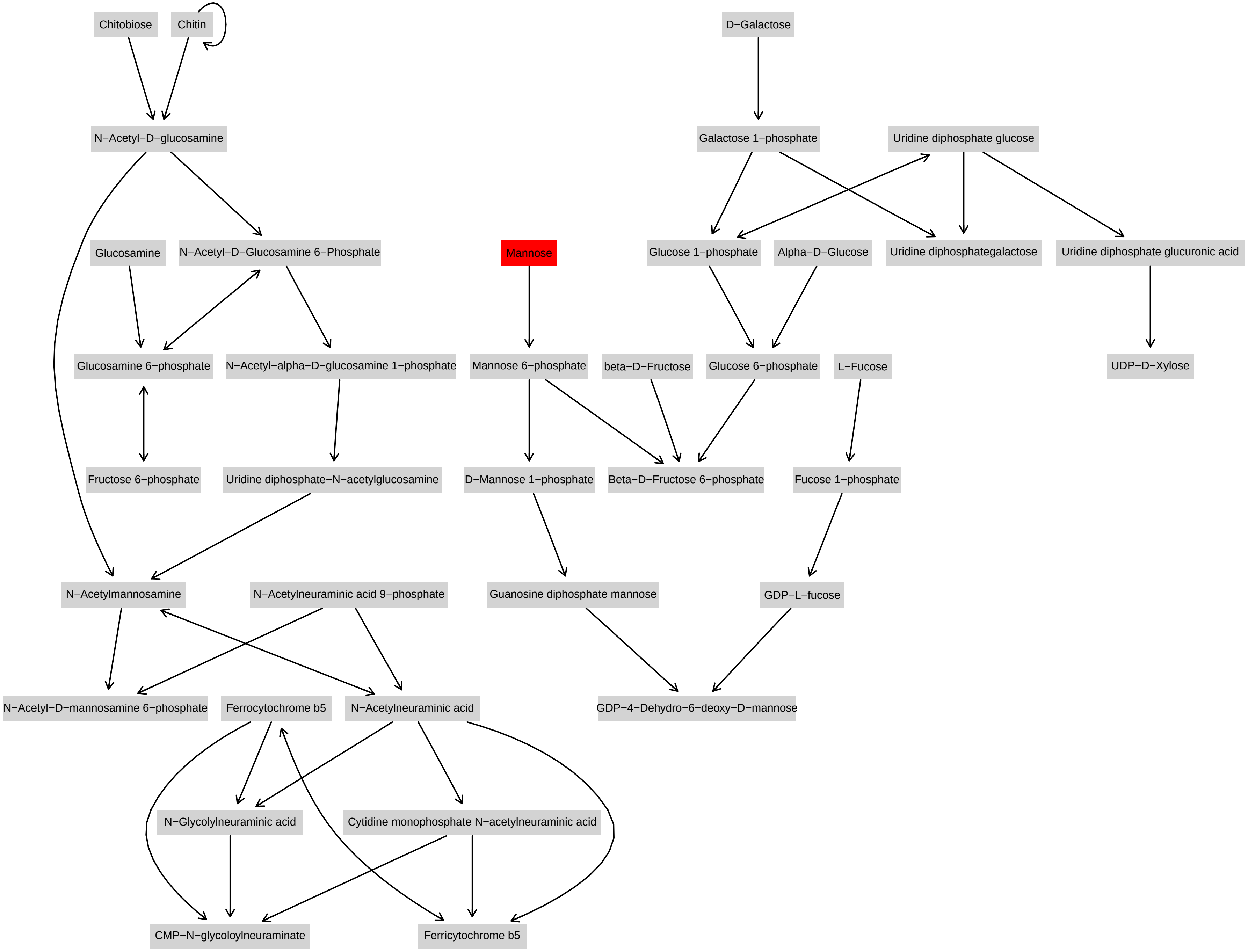

Chitobiose
Chitin
D−Galactose
N−Acetyl−D−glucosamine
Galactose 1−phosphate
Uridine diphosphate glucose
Glucosamine
N−Acetyl−D−Glucosamine 6−Phosphate
Mannose
Glucose 1−phosphate
Alpha−D−Glucose
Uridine diphosphategalactose
Uridine diphosphate glucuronic acid
Glucosamine 6−phosphate
N−Acetyl−alpha−D−glucosamine 1−phosphate
Mannose 6−phosphate
beta−D−Fructose
Glucose 6−phosphate
L−Fucose
UDP−D−Xylose
Fructose 6−phosphate
Uridine diphosphate−N−acetylglucosamine
D−Mannose 1−phosphate
Beta−D−Fructose 6−phosphate
Fucose 1−phosphate
N−Acetylmannosamine
N−Acetylneuraminic acid 9−phosphate
Guanosine diphosphate mannose
GDP−L−fucose
N−Acetyl−D−mannosamine 6−phosphate
Ferrocytochrome b5
N−Acetylneuraminic acid
GDP−4−Dehydro−6−deoxy−D−mannose
N−Glycolylneuraminic acid
Cytidine monophosphate N−acetylneuraminic acid
CMP−N−glycoloylneuraminate
Ferricytochrome b5
